# Nano-Curcumin Prevents Cardiac Injury, Oxidative Stress and Inflammation, and Modulates TLR4/NF-κB and MAPK Signaling in Copper Sulfate-Intoxicated Rats

**DOI:** 10.3390/antiox10091414

**Published:** 2021-09-03

**Authors:** Wedad S. Sarawi, Ahlam M. Alhusaini, Laila M. Fadda, Hatun A. Alomar, Awatif B. Albaker, Amjad S. Aljrboa, Areej M. Alotaibi, Iman H. Hasan, Ayman M. Mahmoud

**Affiliations:** 1 Pharmacology and Toxicology Department, Faculty of Pharmacy, King Saud University, Riyadh 11451, Saudi Arabia; wsarawi@ksu.edu.sa (W.S.S.); aelhusaini@ksu.edu.sa (A.M.A.); lfadda@ksu.edu.sa (L.M.F.); hetalomar@ksu.edu.sa (H.A.A.); abaker@ksu.edu.sa (A.B.A.); 441203053@student.ksu.edu.sa (A.S.A.); 442204129@student.ksu.edu.sa (A.M.A.); ihasan@ksu.edu.sa (I.H.H.); 2 Department of Pharmacology and Toxicology, College of Pharmacy, Umm Al-Qura University, Makkah 21955, Saudi Arabia; 3 Physiology Division, Zoology Department, Faculty of Science, Beni-Suef University, Beni-Suef 62514, Egypt

**Keywords:** curcumin nanoparticles, TLR4, inflammation, cardiotoxicity, DNA damage, oxidative stress

## Abstract

Copper (Cu) is essential for a plethora of biological processes; however, its high redox reactivity renders it potentially toxic. This study investigated the protective effect of curcumin (CUR) and nano-CUR (N-CUR) against Cu cardiotoxicity, emphasizing the role of oxidative stress, TLR4/NF-κB and mitogen-activated protein kinase (MAPK) signaling and cell death in rats. Rats received 100 mg/kg copper sulfate (CuSO_4_), a pesticide used for repelling pests, and were concurrently treated with CUR or N-CUR for 7 days. Cu caused cardiac injury manifested by elevated serum cardiac troponin I (cTnI), creatine kinase (CK)-MB, and lactate dehydrogenase (LDH), as well as histopathological alterations. Cardiac malondialdehyde (MDA), NF-κB p65, TNF-α, and IL-6 were increased, and reduced glutathione (GSH), superoxide dismutase (SOD) and catalase were decreased in Cu-treated rats. CUR and N-CUR prevented cardiac tissue injury, decreased serum cTnI, CK-MB, and LDH, and cardiac MDA, NF-κB p65, TNF-α, and IL-6, and enhanced cellular antioxidants. CUR and N-CUR downregulated TLR4 and AP-1, and decreased the phosphorylation levels of p38 MAPK, JNK, and ERK1/2. In addition, CUR and N-CUR increased cardiac Bcl-2 and BAG-1, decreased Bax and caspase-3, and prevented DNA fragmentation. In conclusion, N-CUR prevents Cu cardiotoxicity by attenuating oxidative injury, inflammatory response, and apoptosis, and modulating TLR4/NF-κB and MAPK signaling. The cardioprotective effect of N-CUR was more potent than the native form.

## 1. Introduction

Copper (Cu) is an essential redox-active trace element found in many tissues. Cu is necessary for the proper functioning of a plethora of biological processes, including blood clotting, antioxidative defense, synthesis of neurotransmitters, protein homeostasis, energy production, and cellular metabolism [1,2]. Cu homeostasis is maintained by regulating its absorption, excretion, and circulating levels through precise regulatory mechanisms [3]. Cu has been established as an environmental pollutant that can harm humans and animals and has been detected in different tissues of aquatic animals [4,5]. The systemic absorption of Cu occurs through different routes, including the gastrointestinal tract, lungs, and skin [6]. Despite its pivotal roles, Cu becomes harmful when the normal limit is surpassed, and chronic exposure has been associated with neurodegenerative disorders, including Alzheimer’s and Parkinson’s diseases [7,8]. In addition, multiorgan dysfunction, such as liver, kidney, and neurological injuries, which could be fatal, are caused by Cu intoxication [9,10]. The liver is the major target for Cu toxicity, and hepatotoxicity is usually seen in individuals with Wilson disease and cirrhosis syndromes [9]. Kidney injury, arrhythmia, seizures, rhabdomyolysis, and intravascular hemolysis are among the clinical manifestations of Cu toxicity [11]. Accordingly, exposure to copper sulfate (CuSO_4_) caused liver and kidney impairment and injury, and hepatic and renal Cu levels were increased in rats [12]. As with other metals, Cu toxicity is managed by chelating agents such as D-penicillamine, tetrathiomolybdate, trientine, and deferoxamine (DFO) [13]. However, the limited or moderate effectiveness and the adverse effects of these chelating agents necessitates the use of safer alternatives.

Cardiotoxicity is myocardial dysfunction and/or damage that could be induced by several drugs and chemicals, including Cu [5,14,15]. Cu caused cardiotoxicity manifested by cellular stress, necrosis, and absence of heartbeat in zebrafish [5,16]. Studies have demonstrated an association between the exposure to high Cu concentrations and the increase in cardiovascular risk and heart failure incidence in humans [17,18,19]. Although the mechanisms underlying Cu toxicity are not fully understood, excess reactive oxygen species (ROS), oxidative stress [20,21], and endocrine perturbation [22] have been implicated. ROS are versatile oxidants that damage several cellular macromolecules, including lipids, proteins, and DNA, resulting in cell death [23,24]. Oxidative stress has been demonstrated in the hippocampus and frontal cortex of rats that received CuSO_4_ [25]. Impairment of memory and learning was associated with oxidative stress in the hippocampus of rats treated with Cu chloride [10]. These studies demonstrated the significant role of oxidative stress in Cu toxicity. Hence, suppression of oxidative stress can protect against cardiotoxicity and other deleterious effects of Cu.

Curcumin (CUR) is a hydrophobic polyphenol found in turmeric [26] and possesses potent antioxidant [27], anti-inflammatory [28,29], and cardioprotective effects [30]. In a rat model of lung injury, CUR suppressed inflammation, oxidative stress, and apoptosis and enhanced antioxidant defenses [28]. The anti-inflammatory and antioxidant properties of CUR were demonstrated in a rat model of lipopolysaccharide (LPS)/diclofenac-induced liver injury [29]. CUR attenuated oxidative stress and inflammatory response and prevented tissue injury in experimental models of hepatotoxicity and nephrotoxicity induced by lead [31] and gentamicin [32,33]. In addition to boosting antioxidants, the therapeutic effect of CUR was associated with modulating toll-like receptor (TLR)-4/nuclear factor-kappaB (NF-κB) and mitogen-activated protein kinase (MAPK) signaling in rat liver [29]. TLRs are members of the interleukin (IL)-1 receptor family, and TLR4 is expressed in the heart. These receptors enable cardiomyocytes to respond to endogenous or exogenous signals. Oxidative stress is one of the endogenous signals that activate TLRs and contribute to congestive heart failure (HF) [34]. Upon activation, TLR4 triggers the release of pro-inflammatory mediators through promoting NF-κB and MAPKs [35]. TLR4 is activated in several cardiac alterations, such as cardiotoxicity, cardiomyopathy, and HF [36]. However, the possible involvement of TLR4 signaling in Cu cardiotoxicity has not been demonstrated. This study investigated the effect of Cu exposure on cardiac TLR4/NF-κB and MAPK signaling in rats, and the possible protective role of CUR. Owing to the poor systemic bioavailability and rapid metabolism of CUR, the main drawbacks limiting its therapeutic uses [37], we aimed to investigate the protective effect of nano-CUR (N-CUR).

## 2. Materials and Methods

### 2.1. Chemicals and Reagents

N-CUR was obtained from Lipolife (Essex, UK) and DFO was supplied by Novartis Pharma AG (Rotkreuz, Switzerland). CuSO_4_, CUR, primers, carboxymethylcellulose (CMC), sodium dodecyl sulfate (SDS), agarose, pyrogallol, thiobarbituric acid (TBA), and reduced glutathione (GSH) were obtained from Sigma (St. Louis, MO, USA). Caspase-3 assay kit, NF-κB p65, cardiac troponin I (cTnI), Bax and Bcl-2 ELISA kits were supplied by MyBiosource (San Diego, CA, USA), and TNF-α and IL-6 ELISA kits were supplied by R&D Systems (Minneapolis, MN, USA). Creatine kinase (CK)-MB and lactate dehydrogenase (LDH) kits were purchased from Spinreact (Girona, Spain). Antibodies against TLR4, *p*-p38 MAPK, p38 MAPK, pJNK, JNK, pERK1/2, ERK1/2, and β-actin were supplied by Novus Biologicals (Centennial, CO, USA). Other chemicals and kits were supplied by standard manufacturers.

### 2.2. Animals and Treatments

Male Wistar rats (180–200 g), obtained from the Animals Care Centre at King Saud University, were housed under standard conditions and 12 h light/dark cycle and given free access to food and water. The animals were acclimatized for one week before starting the experiment.

After acclimatization, 40 rats were randomly allocated into 5 groups (*n* = 8) as follows:

Group I (Control): received the vehicle.

Group II (CuSO_4_): received 100 mg/kg CuSO_4_ [12,38].

Group III (DFO): received DFO (23 mg/kg) [39] and 100 mg/kg CuSO_4_.

Group IV (CUR): received 80 mg/kg CUR [6,39] and 100 mg/kg CuSO_4_.

Group V (N-CUR): received 80 mg/kg N-CUR [6,39] and 100 mg/kg CuSO_4_.

CuSO_4_, DFO, CUR and N-CUR were dissolved in 1% CMC and supplemented via oral gavage for 7 days. The rats were sacrificed under ketamine/xylazine anesthesia, blood was collected, and the heart was removed, washed in cold phosphate buffered saline (PBS), and the parts of left ventricle were kept frozen in liquid nitrogen. Other samples from the heart were fixed in 10% neutral buffered formalin (NBF) while others were homogenized in 10 mM ice-cold Tris–HCl buffer (pH 7.4), centrifuged at 6000 rpm for 10 min and the clear supernatant was used for the determination of malondialdehyde (MDA), GSH, superoxide dismutase (SOD), catalase (CAT), Bax, Bcl-2, caspase-3, TNF-α, IL-6 and NF-κB p65.

### 2.3. Determination of Cardiac Injury, MDA, and Antioxidants

Serum cTnI was assayed using ELISA kit (MyBiosource, San Diego, CA, USA), and CK-MB and LDH were determined using reagent kits supplied by Spinreact (Girona, Spain), following the manufacturers’ instructions.

Cardiac MDA was assayed as previously described [40]. Briefly, the heart tissue homogenate was mixed with TBA, SDS, and acetate buffer, and heated in boiling water bath for 60 min. After cooling, *n*-butanol was added to the mixture, which was centrifuged, and absorbance of the organic layer was measured at 532 nm. GSH was determined according to the method of Ellman [41]. This assay is based on the reaction of GSH with 5,5′-dithio-bis (2-nitrobenzoic acid) and measurement of the absorbance of the yellow-colored product at 412 nm. Given its ability to inhibit pyrogallol autoxidation, SOD activity was determined following the method of Marklund and Marklund [42]. The activity of CAT was determined in the heart homogenate by monitoring the decomposition of hydrogen peroxide according to the method of Cohen et al. [43].

### 2.4. Determination of NF-κB p65, Cytokines, Apoptosis Markers, and DNA Fragmentation

Cardiac NF-κB p65, Bax and Bcl-2 were assayed using specific ELISA kits (MyBioSource, San Diego, CA, USA), and TNF-α and IL-6 were assayed using R&D Systems (Minneapolis, MN, USA) ELISA kits. Caspase-3 was determined in the heart homogenate using assay kit supplied by MyBioSource (San Diego, CA, USA). All assays were conducted according to the instructions of the manufacturers. Agarose electrophoresis and the colorimetric methods [44] were used to assess DNA fragmentation. The results were presented as a fold change of the control.

### 2.5. Histological Examination

The heart samples, fixed for 24 h in 10% NBF, were dehydrated, embedded in paraffin, and cut into 5-µm thick-sections. The prepared sections were processed for staining with hematoxylin and eosin (H&E) and were examined using a light microscope.

### 2.6. Gene Expression

The effect of CuSO_4_ and treatment agents on the expression of cardiac activator protein-1 (AP-1) and BCL2 associated Athanogene (BAG)-1 were determined by qRT-PCR as previously described [45]. Briefly, RNA was isolated from the heart samples using TRIzol (ThermoFisher Scientific, Waltham, MA, USA), treated with RNase-free DNase (Qiagen, Hilden, Germany), quantified using a nanodrop, and samples with OD260/OD280 nm ratio of ≥1.8 were reverse transcribed into cDNA. Amplification of cDNA was carried out using SYBR green master mix (ThermoFisher Scientific, Waltham, MA, USA) and the primers in Table 1 in a total reaction volume of 20 μL. The obtained data were analyzed using the 2^−ΔΔCt^ method [46] and normalized to β-actin.

### 2.7. Western Blotting

The frozen heart samples were homogenized in RIPA buffer supplemented with proteinase/phosphatase inhibitors, centrifuged, and the supernatant was collected. Protein concentration in the supernatant was assayed using Bradford protein assay kit (BioBasic, Markham, Canada), and 50 µg protein was subjected to 10% SDS/PAGE and electro-transferred to nitrocellulose membranes. The membranes were blocked in 5% milk in TBST followed by incubation overnight at 4 °C with primary antibodies against TLR4, *p*-p38 MAPK, p38 MAPK, pJNK, JNK, pERK1/2, ERK1/2, and β-actin. The membranes were washed, probed with secondary antibodies, washed with TBST, and developed using Clarity™ Western ECL Substrate from BIO-RAD (Hercules, CA, USA). The bands were visualized in ImageQuant LAS 4000 and quantified using ImageJ (version 1.32j, NIH, USA).

### 2.8. Statistical Analysis

The results are expressed as mean ± standard deviation (SD). Statistical analysis and multiple comparisons were performed by one-way analysis of variance (ANOVA) and Tukey’s post-hoc test using GraphPad Prism 8. A *p* value < 0.05 was considered significant.

## 3. Results

### 3.1. N-CUR and CUR Prevent Cu-Induced Cardiac Injury in Rats

**Figure 1:**
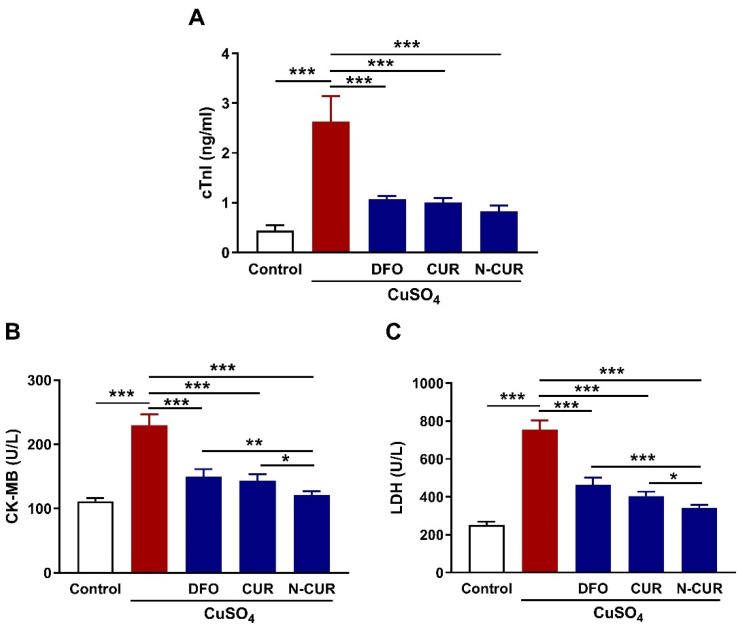
N-CUR and CUR prevent Cu-induced cardiac injury in rats. Treatment with N-CUR, CUR, and DFO ameliorated serum cardiac troponin I (cTnI) (**A**), creatine kinase (CK)-MB (**B**) and lactate dehydrogenase (LDH) (**C**). Data are mean ± SD, (*n* = 8). * *p* < 0.05, ** *p* < 0.01 and *** *p* < 0.001.

The cardioprotective effect of CUR and N-CUR against Cu toxicity was evaluated through the assessment of circulating cardiac function markers (cTnI, CK-MB, and LDH) and histopathological investigation. Cu-exposed rats exhibited significantly elevated serum cTnI (Figure 1A), CK-MB (Figure 1B) and LDH (Figure 1C) as compared to the control rats (*p* < 0.001). Treatment of the Cu-exposed rats with DFO, CUR, or N-CUR ameliorated serum cTnI, CK-MB, and LDH significantly (*p* < 0.001). The effect of N-CUR on serum CK-MB and LDH was significant when compared with DFO (*p* < 0.01; *p* < 0.001) and CUR (*p* < 0.05; *p* < 0.05).

**Figure 2:**
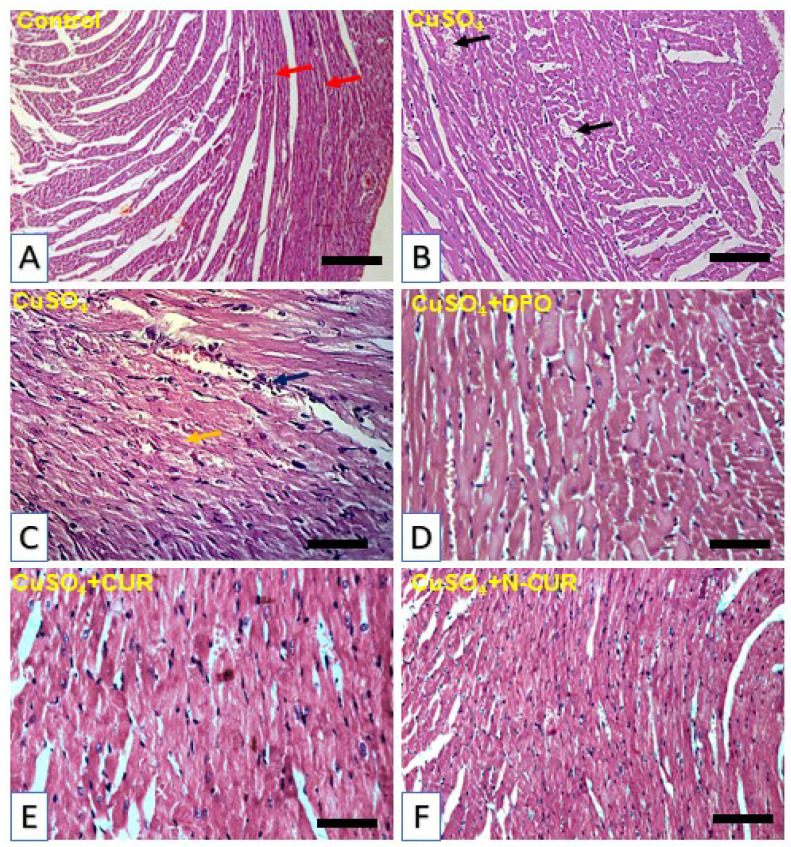
Photomicrographs of H&E-stained sections in the heart of (**A**) control rats showing normal histological structure of the cardiomyocytes (red arrow), (**B**,**C**) CuSO_4_-intoxicated rats showing blood vessel congestions (black arrow), inflammatory cell infiltration (blue arrow) and degenerative changes (yellow arrow), (**D**,**F**) CuSO_4_-intoxicated rats treated with deferoxamine (DFO) (**D**), curcumin (CUR) (**E**), and nano-CUR (N-CUR) (**F**) showing no histopathological alterations. (Scale bar = 50 µm, −400X).

The histopathological investigation showed normal morphological appearance with normal cardiomyocytes in the control group (Figure 2A). In contrast, Cu administration induced the appearance of blood vessel congestions, inflammatory cells infiltration and degenerative changes as shown in Figure 2B,C. In parallel with the biochemical findings, sections in the heart of Cu-exposed rats treated with DFO (Figure 2D), CUR (Figure 2E), and N-CUR (Figure 2F) showed no histopathological alterations.

### 3.2. N-CUR and CUR Attenuate Cardiac Oxidative Stress in Cu-Induced Rats

**Figure 3:**
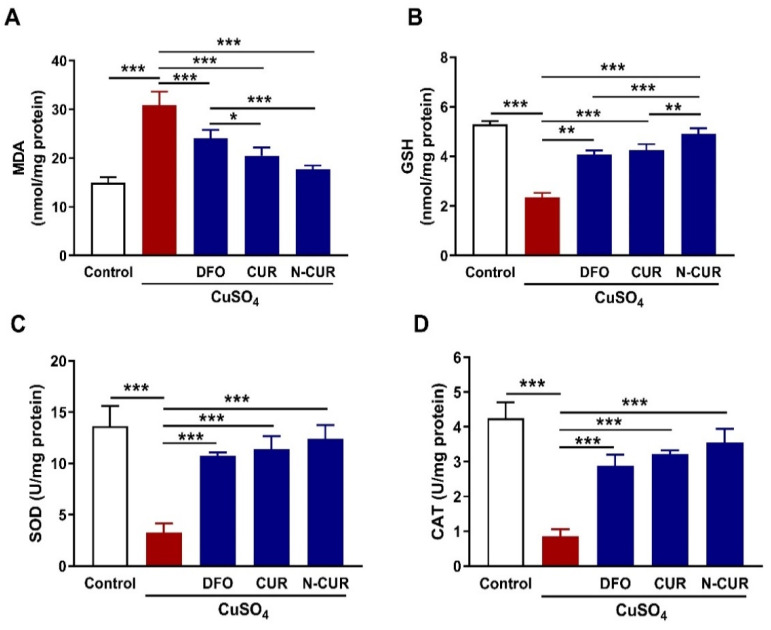
N-CUR and CUR attenuate cardiac oxidative stress in Cu-intoxicated rats. Treatment with N-CUR, CUR, and DFO decreased cardiac MDA (**A**) and increased GSH (**B**), SOD (**C**), and CAT (**D**). Data are mean ± SD, (*n* = 8). * *p* < 0.05, ** *p* < 0.01 and *** *p* < 0.001.

Cardiac lipid peroxidation (LPO), assessed as MDA, was significantly elevated in Cu-intoxicated rats when compared with the control group (*p* < 0.001; Figure 3A). The content of the cellular antioxidant GSH (Figure 3B), as well as the activities of SOD (Figure 3C) and CAT (Figure 3D), were markedly decreased in the heart of Cu-intoxicated rats (*p* < 0.001). DFO, CUR, and N-CUR decreased MDA and increased GSH and SOD in the heart of Cu-treated rats. The effect of N-CUR on cardiac MDA and GSH was significant as compared to DFO (*p* < 0.001; *p* < 0.001) and CUR (*p* < 0.05; *p* < 0.01).

### 3.3. N-CUR and CUR Suppress Cardiac TLR4/NF-κB and MAPK Signaling in Cu-Induced Rats

**Figure 4:**
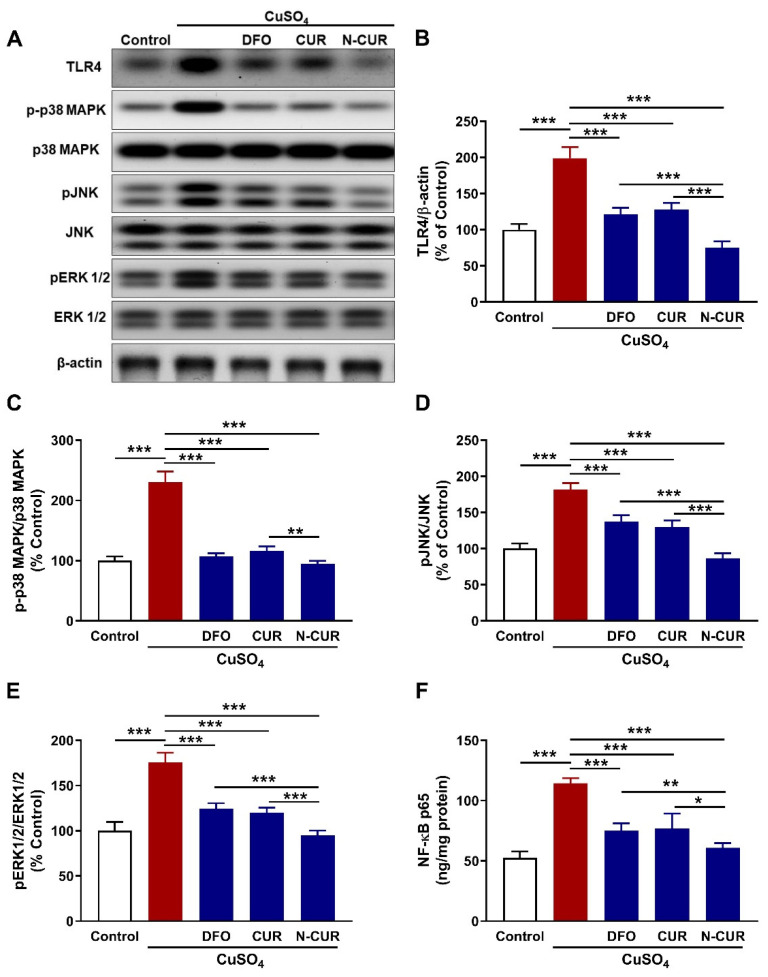
N-CUR and CUR suppress cardiac TLR4/NF-κB and MAPK signaling in Cu-intoxicated rats. (**A**) Representative blots showing changes in the expression of TLR4, p38 MPAK, JNK, ERK1/2, and β-actin. (**B**–**F**) N-CUR, CUR, and DFO downregulated cardiac TLR4 (**B**) and decreased the phosphorylation levels of p38 MAPK (**C**), JNK (**D**), ERK1/2 (**E**), and NF-κB (**F**). Data are mean ± SD, (*n* = 8). * *p* < 0.05, ** *p* < 0.01 and *** *p* < 0.001.

The protein expression of TLR4 was significantly upregulated in the heart of rats that received Cu (*p* < 0.001) as depicted in Figure 4A,B. Treatment with DFO, CUR, and N-CUR significantly downregulated TLR4 in the heart of Cu-exposed rats. N-CUR downregulated cardiac TLR4 expression significantly when compared with DFO or CUR.

To evaluate changes in the intracellular signaling in Cu-exposed rats and the modulatory role of DFO, CUR, and N-CUR, the phosphorylation levels of p38 MAPK, JNK, and ERK1/2 were determined using Western blotting. Cu increased the phosphorylation levels of p38 MAPK (Figure 4C), JNK (Figure 4D) and ERK1/2 (Figure 4E) in the heart of rats significantly as compared to the control animals (*p* < 0.001). All treatment agents effectively decreased the phosphorylation levels of cardiac p38 MAPK, JNK, and ERK1/2 in Cu-intoxicated rats (*p* < 0.001). N-CUR decreased the phosphorylation of p38 MAPK significantly when compared with CUR (*p* < 0.01), and pJNK (*p* < 0.001) and pERK1/2 (*p* < 0.001) when compared with either DFO or CUR.

Additionally, NF-κB p65 was significantly increased in the heart of Cu-treated rats (*p* < 0.001) and decreased in rats that received DFO, CUR, or N-CUR as represented in Figure 4F. When compared with DFO or CUR, N-CUR was more effective in downregulating cardiac NF-κB p65 in Cu-intoxicated rats (*p* < 0.01 and *p* < 0.05, respectively).

### 3.4. N-CUR and CUR Mitigate Cardiac AP-1, TNF-α, and IL-6 in Cu-Induced Rats

**Figure 5:**
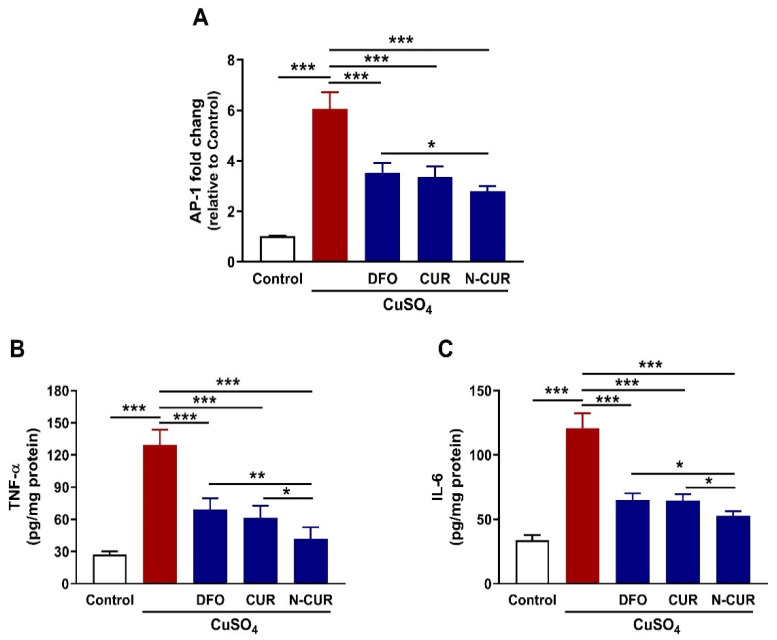
N-CUR and CUR downregulated cardiac AP-1 mRNA abundance (**A**), TNF-α (**B**) and IL-6 (**C**) in Cu-intoxicated rats. Data are mean ± SD, (*n* = 8). * *p* < 0.05, ** *p* < 0.01 and *** *p* < 0.001.

Exposure of the rats to Cu increased AP-1 mRNA abundance in the heart significantly (*p* < 0.001) as compared to the control rats (Figure 5A). In the same context, cardiac levels of TNF-α (*p* < 0.001) and IL-6 (*p* < 0.001) were increased in Cu-exposed rats as represented in Figure 5B,C, respectively. Treatment of the Cu-exposed rats with DFO, CUR, or N-CUR downregulated cardiac AP-1 mRNA and decreased TNF-α and IL-6 (*p* < 0.001). N-CUR decreased AP-1 significantly when compared with DFO (*p* < 0.01), and TNF-α and IL-6 as compared with either DFO (*p* < 0.01 and *p* < 0.05) or CUR (*p* < 0.05 and *p* < 0.05).

### 3.5. N-CUR and CUR Prevent Apoptosis in Cu-Intoxicated Rats

**Figure 6:**
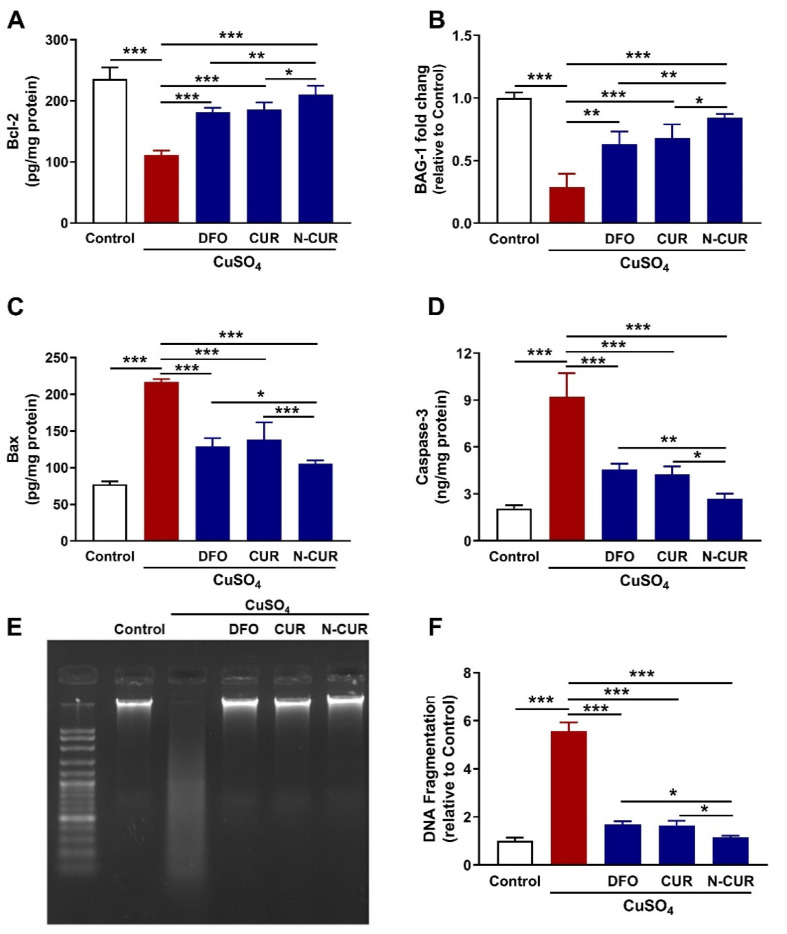
N-CUR and CUR prevent apoptosis in Cu-intoxicated rats. N-CUR, CUR, and DFO increased cardiac Bcl-2 (**A**) and BAG-1 mRNA (**B**), decreased Bax (**C**), and caspase-3 (**D**), and prevented DNA fragmentation (**E**,**F**) in Cu-intoxicated rats. Data are mean ± SD, (*n* = 8). * *p* < 0.05, ** *p* < 0.01 and *** *p* < 0.001.

The expression levels of Bcl-2, BAG-1, Bax, and caspase-3 were assessed to evaluate the protective effect of CUR, and N-CUR on Cu-induced cardiomyocyte apoptosis. Bcl-2 (Figure 6A) and BAG-1 (Figure 6B) were significantly downregulated in the heart of Cu-intoxicated rats as compared to the control group (*p* < 0.001). DFO, CUR, and N-CUR upregulated cardiac Bcl-2 protein and BAG-1 mRNA abundance in Cu-treated rats. Bax (Figure 6C) and caspase-3 (Figure 6D) showed significant increase in the heart of rats exposed to Cu as compared to the control group (*p* < 0.001), effects that were reversed in rats that received DFO, CUR, or N-CUR. The effect of N-CUR on Bcl-2, BAG-1, Bax, and caspase-3 was significant when compared with either CUR or DFO.

The protective effect of CUR and N-CUR against Cu-induced cardiomyocyte cell death was further confirmed via assessment of DNA fragmentation (Figure 6E,F). Cu-intoxicated rats showed an increase in DNA fragmentation levels as compared to the control group (*p* < 0.001). All treatments (DFO, CUR, and N-CUR) prevented the deleterious effect of Cu on DNA integrity. N-CUR effectively decreased DNA fragmentation when compared with CUR or DFO (*p* < 0.05).

## 4. Discussion

Copper is an essential element for a plethora of cellular processes and plays a key role as a catalytic factor for many enzymes, such as SOD and cytochrome c oxidase [2]. Cu is present in both oxidized and reduced forms and its high redox reactivity makes it a source of ROS and makes it potentially toxic [20]. Cu can disturb the redox homeostasis, and provoke oxidative stress and cellular damage, leading to several diseases. Hepatotoxicity, kidney injury, and neurodegenerative disorders were associated with the exposure to Cu [7,8,9,10,12]; however, the toxic effects of Cu on the heart are less documented. Herein, we evaluated the possible involvement of oxidative stress, and TLR4/NF-κB and MAPK signaling, in the cardiotoxic effect of Cu in rats, and the protective effect of CUR and N-CUR.

Exposure to CuSO_4_ caused cardiac injury manifested by the elevated circulating cTnI, CK-MB, and LDH. cTnI is a highly specific and sensitive marker for myocardial injury, and increased serum CK-MB and LDH denotes myocardial damage [47]. In support of the biochemical findings, the histopathological examination revealed congestions, degenerative changes, and inflammatory cell infiltrations in the myocardium of CuSO_4_-treated rats. CuSO_4_ is a pesticide used for repelling pests in agriculture and for minimizing contamination risk in tissue culture incubators due to its bactericidal and fungicidal properties [48]. Accidental intoxication among farm workers exposed to CuSO_4_ has been reported, and toxic levels of this pesticide can lead to methemoglobinemia and death [48]. Cardiotoxicity due to exposure to Cu has been reported in zebrafish [5,16], and increased incidence of HF in human was associated with high Cu levels [17,18,19]. Owing to its ability to generate ROS, cardiotoxicity of Cu could be directly attributed to oxidative stress. The present study showed an increase in cardiac LPO and decreased GSH and antioxidant enzymes (SOD and CAT), demonstrating an oxidative stress status. The redox nature of Cu renders it toxic due to the generation of highly reactive hydroxyl radicals (•OH) [20]. In addition, Cu can alter the activity of respiratory chain enzymes resulting in increased mitochondrial ROS generation [21]. ROS are potent oxidizing agents that provoke oxidative damage of cellular macromolecules and cell death [23,24]. Accordingly, oxidative stress has been reported in the hippocampus of rats following the administration of CuSO_4_ [25] and Cu chloride [10]. The current study introduced information on the involvement of oxidative stress in CuSO_4_ cardiotoxicity in rats.

CUR and N-CUR ameliorated serum cTnI, CK-MB, and LDH, and prevented all histopathological alterations in the heart of CuSO_4_-intoxicated rats. Since oxidative stress plays the key role in Cu toxicity, the cardioprotective effect of CUR is a direct consequence of its radical-scavenging and antioxidant properties. Here, rats that received CUR and N-CUR exhibited remarkable reduction in cardiac LPO and enhanced GSH, SOD, and CAT. The antioxidant activity of CUR has been reported in several studies of experimental cardiotoxicity induced by doxorubicin, daunorubicin, cisplatin, cyclophosphamide, irinotecan, bisphenol, atrazine, and isoproterenol (reviewed in [49]). CUR protected rats against liver and kidney injury induced by CuSO_4_ through the suppression of MDA and enhancement of antioxidants [6], and prevented CuSO_4_-induced oxidative stress in *Drosophila melanogaster* [50]. Besides its radical-scavenging properties, CUR can activate Nrf2, which is a redox-sensitive factor that regulates antioxidant genes and suppresses oxidative stress [51]. CUR is a potent Nrf2 activator, and studies have shown upregulation of Nrf2-dependent antioxidant genes following its administration [29,52,53].

Besides attenuation of oxidative stress, CUR and N-CUR suppressed the inflammatory response elicited by CuSO_4_ in the heart of rats. CuSO_4_ promoted the activation of TLR4/NF-κB and MAPK signaling in the heart of rats. Consequently, an increase in the levels of pro-inflammatory cytokines (TNF-α and IL-6) was reported, pinpointing cardiomyocyte inflammation. Activation of TLR4 signaling in the heart of Cu-intoxicated rats is a direct consequence of excessive ROS generation and cell injury [34]. Activation of TLR4 occurs in cardiotoxicity, cardiomyopathy, HF, and other cardiac alterations [36]. TLR4 triggers both the MyD88-dependent and -independent pathways, leading to the activation of transcription factors such as NF-κB and the production of pro-inflammatory mediators [35]. MyD88 recruits and activate IL-1 receptor-associated kinases (IRAKs), which in turn activate TNF receptor-associated factor 6 (TRAF6), and then TAK1 is activated [54]. TAK1 phosphorylates both MAPKs and the IKK complex. It induces I*κ*B phosphorylation leading to NF-κB activation and translocation into the nucleus to promote the expression of pro-inflammatory cytokines. TAK1 also activates p38 MAPK, JNK, and ERK1/2 and the activated MAPK signaling activates the transcription factor AP-1, which contributes to the release of pro-inflammatory cytokines [54]. In the present study, TLR4 was upregulated, and the phosphorylation levels of NF-κB, p38 MAPK, JNK, and ERK1/2 were increased in the heart of CuSO_4_-induced rats. Consequently, the expression of TNF-α and IL-6 was upregulated.

CUR and N-CUR effectively suppressed the cardiac inflammatory response triggered by CuSO_4_ in rats, demonstrating a potent anti-inflammatory activity. CUR can effectively inhibit TLR4 homodimerization, which is the initial step in activating the inflammatory response [55]. This supported the suppression of TLR4 signaling in the heart of CuSO_4_-induced rats treated with CUR. CUR has anti-inflammatory activities and downregulates MAPKs, TNF-α, and IL-6 [55]. It can inhibit both MyD88- and TRIF-dependent pathways [55], and computational approaches have revealed that it can fit into the myeloid differentiation factor 2 (MD2) pocket [56]. Given the role of oxidative stress in activating TLR4, the ameliorative effect of CUR could be attributed to its ability to suppress both oxidative stress and TLR4-mediated inflammatory response. The anti-inflammatory effect of CUR has also been supported by studies employing experimental models of cardiotoxicity [49].

CUR and N-CUR prevented apoptotic cell death in the heart of CuSO_4_-induced rats, adding support to their cardioprotective activities. Apoptotic cell death was observed in the heart of Cu-administered rats in the present study where Bcl-2 and BAG-1 were downregulated, and Bax and caspase-3 were upregulated. Cell death due to Cu intoxication could be directly attributed to the provoked oxidative stress and inflammatory response. Cu-mediated ROS can induce mitochondrial permeability transition in different cells, including astrocytes [57] and hepatocytes [58], leading to apoptotic cell death. In addition, surplus ROS can activate the pro-apoptotic protein Bax, which induce the release of mitochondrial cytochrome *c* by promoting the loss of mitochondrial membrane potential via voltage-dependent anion channel [59]. Through interaction with Apaf, cytochrome *c* can initiate the activation cascade of caspases. Caspase-3 is the executioner caspase that elicits DNA fragmentation or degradation of cytoskeletal proteins, culminating in cell death [60]. The active caspase-3 itself favors further release of cytochrome *c* from the mitochondria and amplification of the death signal [60]. Accordingly, analysis of DNA fragmentation revealed a significant increase in the heart of CuSO_4_-intoxicated rats. In contrast, Bcl-2 and BAG-1 were decreased in the heart of rats that received CuSO_4_. Bcl-2 suppresses the release of cytochrome *c* and prevents apoptosis [61], and BAG-1 is a multifunctional protein that synergizes the action of Bcl-2 to suppress cell death [62]. CUR upregulated the anti-apoptotic factors Bcl-2 and BAG-1, suppressed Bax and caspase-3, and prevented DNA fragmentation, demonstrating a potent anti-apoptotic effect, which is a direct consequence of its antioxidant and anti-inflammatory properties.

N-CUR exerted a significant modulatory effect on cardiac function markers, LPO, GSH, TLR4, pro-inflammatory cytokines, phosphorylation levels of NF-κB, p38 MAPK, JNK, and ERK1/2, apoptosis markers, and DNA fragmentation when compared with CUR. The superior effect of N-CUR is a direct result of the improved properties of CUR. Despite its reported pharmacological effects, the therapeutic applications of CUR are limited by its poor water solubility, absorption and systemic bioavailability, rapid metabolism, physicochemical instability, and low penetration and targeting efficacy [37]. This notion is supported by studies showing the stronger antioxidant and anti-inflammatory properties of N-CUR. For instance, N-CUR displayed stronger radical-scavenging and anti-LPO properties than the native compound in hepatoma cells lines [63]. CUR nanoparticles showed better solubility and downregulated NF-κB and pro-inflammatory mediators in LPS-challenged macrophages than the native form [64].

## 5. Conclusions

**Figure 7:**
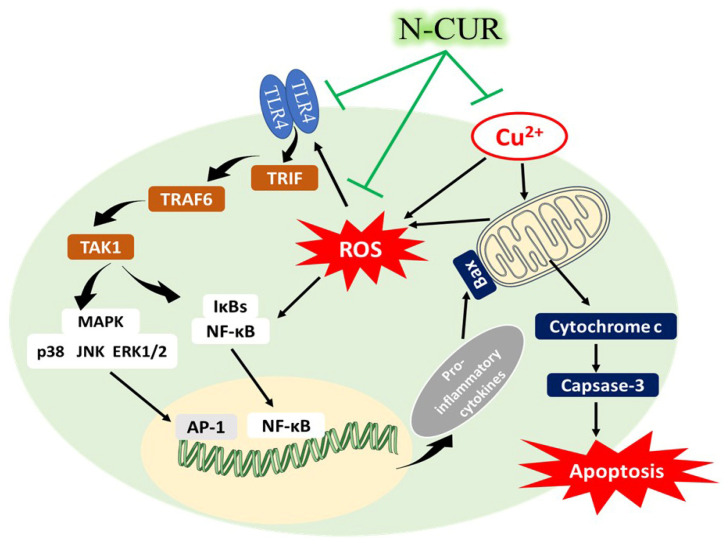
A schematic diagram illustrating the cardiotoxic effect of Cu and the protective effect of N-CUR. Cu elicits the generation of ROS and activates TLR4, NF-κB and MAPKs, leading to the release of pro-inflammatory cytokines and apoptotic cell death. N-CUR prevented Cu-induced cardiac tissue injury by suppressing oxidative stress, inflammation and cell death, effects that were associated with suppression of TLR4/NF-κB and MAPKs signaling.

These results confer information on the cardiotoxic effect of Cu and the protective effect of CUR and its nanoform. N-CUR and CUR ameliorated cardiac function markers, and attenuated cardiac tissue injury, oxidative stress, inflammation, and cell death in Cu-intoxicated rats. These beneficial effects were associated with suppression of TLR4/NF-κB and MAPKs signaling (Figure 7). N-CUR exerted a stronger cardioprotective efficacy as compared to the native form, an effect that could be explained in terms of improved properties of CUR. Therefore, N-CUR can confine the cardiotoxicity of CuSO_4_ by attenuating oxidative damage, inflammation, and apoptosis, pending further studies to explore other involved mechanisms.

## Figures and Tables

**Table 1:** Primers used for qRT-PCR.

Gene	GenBank Accession Number	Primer Sequence (5′-3′)
*AP-1*	NM_021835.3	F: TGGGCACATCACCACTACAC R: GGGCAGCGTATTCTGGCTAT
*BAG1*	NM_001106647.3	F: GGTCCAGACGGAGGAAATGG R: ACTGTTACCTTGCTGTGGGG
*β-actin*	NM_031144.3	F: AGGAGTACGATGAGTCCGGC R: CGCAGCTCAGTAACAGTCCG

## Data Availability

Data analyzed or generated during this study are included in this manuscript.
